# Triphenylphosphonium-Functionalized Gold Nanorod/Zinc Oxide Core–Shell Nanocomposites for Mitochondrial-Targeted Phototherapy

**DOI:** 10.3390/pharmaceutics16020284

**Published:** 2024-02-16

**Authors:** Ara Joe, Hyo-Won Han, Yu-Ra Lim, Panchanathan Manivasagan, Eue-Soon Jang

**Affiliations:** Department of Applied Chemistry, Kumoh National Institute of Technology, Gumi 730-701, Gyeongbuk, Republic of Korea; jar@kumoh.ac.kr (A.J.); 20101414hyowon@kumoh.ac.kr (H.-W.H.); tig02268@naver.com (Y.-R.L.) manimaribtech@kumoh.ac.kr (P.M.)

**Keywords:** gold nanorods, zinc oxide, triphenylphosphonium, cancer, phototherapy

## Abstract

Phototherapies, such as photothermal therapy (PTT) and photodynamic therapy (PDT), combined with novel all-in-one light-responsive nanocomposites have recently emerged as new therapeutic modalities for the treatment of cancer. Herein, we developed novel all-in-one triphenylphosphonium-functionalized gold nanorod/zinc oxide core–shell nanocomposites (CTPP-GNR@ZnO) for mitochondrial-targeted PTT/PDT owing to their good biocompatibility, tunable and high optical absorption, photothermal conversion efficiency, highest reactive oxygen species (ROS) generation, and high mitochondrial-targeting capability. Under laser irradiation of 780 nm, the CTPP-GNR@ZnO core–shell nanocomposites effectively produced heat in addition to generating ROS to induce cell death, implying a synergistic effect of mild PTT and PDT in combating cancer. Notably, the in vitro PTT/PDT effect of CTPP-GNR@ZnO core–shell nanocomposites exhibited effective cell ablation (95%) and induced significant intracellular ROS after the 780 nm laser irradiation for 50 min, indicating that CTPP in CTPP-GNR@ZnO core–shell nanocomposites can specifically target the mitochondria of CT-26 cells, as well as generate heat and ROS to completely kill cancer cells. Overall, this light-responsive nanocomposite-based phototherapy provides a new approach for cancer synergistic therapy.

## 1. Introduction

Cancer is one of the greatest global threats to human health [1,2]. Current cancer therapies such as radiation therapy, chemotherapy, and surgery play a crucial role in cancer treatment [3,4]. However, these conventional therapeutic methods are only partially successful, with certain drawbacks such as causing severe toxic side effects to normal cells and tissues, increasing the incidence of second cancers, damaging the immune system, inducing drug resistance, and having a limited specificity, which can ultimately influence the survival rates of cancer patients [5,6]. Therefore, developing novel therapeutic modalities for more effective cancer treatment is urgently required. 

Alternatively, phototherapies such as photothermal therapy (PTT) and photodynamic therapy (PDT) have emerged as new therapeutic modalities for the treatment of cancer owing to their many advantages, such as minimal invasiveness, target specificity, high efficiency, deeper tissue penetration, remote-control capabilities, and minimal side effects [7,8]. PTT using photo-absorbing nanomaterials converts near-infrared (NIR) light to heat (>43 °C), which thermally burns the cancer cells and tissues and is a highly effective therapy owing to its ease of operation, rapid recovery, and short treatment time [9]. PDT is another therapeutic modality that uses light irradiation to activate a photosensitizer to generate reactive oxygen species (ROS), including singlet oxygen (^1^O_2_), hydroxyl radical (•OH), and superoxide (O_2_^−^•) to kill cancer cells [10]. The two (PTT/PDT) aforementioned methods can cause irreversible cancer cell damage, leading to cell death via protein and enzyme denaturation, cancer cell membrane destruction, nuclear damage, and endoplasmic reticulum dysfunction [11,12,13]. Nevertheless, a single therapy (PTT alone or PDT alone) has inherent limitations [13]. For example, PTT alone requires a high temperature (>50 °C) to kill cancer cells, which cannot maximize the therapeutic effect owing to the uneven heat distribution in large tumors [14,15]. A high laser power density and high temperature would damage the surrounding healthy cells and tissues during PTT [14]. Therefore, a mild temperature during PTT (~45 °C) should be more practical in clinical settings [16,17]. Regarding PDT, the hypoxic microenvironment of solid tumors significantly limits the effectiveness of the treatment [18]. Several studies have reported that a combination of PTT using mild temperatures along with PDT has recently emerged as an intriguing alternative to a single therapy alone owing to the synergistic effect of the treatment, which can successfully eliminate cancer cells [14,19]. Therefore, light-activated all-in-one nanomaterial-based mild PTT and PDT are highly desired, and remains an emerging task in medical therapies [20]. 

A variety of nanomaterials have been designed to cure cancer cells and tumor tissues as PTT agents, such as gold nanorods (GNRs), gold nanoshells, gold nanoplates, gold nanocages, silver nanoparticles (NPs), platinum NPs, copper sulfide NPs, palladium NPs, iron sulfide NPs, polypyrrole NPs, and carbon NPs [21,22]. Among these, GNRs have been demonstrated to be significantly promising as highly efficient PTT agents owing to their outstanding properties, such as photothermal conversion efficiency, high absorption efficiency, surface modification, biocompatibility, easy synthesis, easy tunability, controllable size, and low toxicity, thereby resulting in a better enhancement of the heat generation rate compared to other NPs [23,24]. Currently, the classic seed-mediated growth method has proven to be the most successful in controlling the shape of high-quality GNRs and is easier than the synthesis of gold nanoshells, gold nanoplates, and gold nanocages [25,26]. GNRs exhibit two localized surface plasmon resonance (LSPR) modes: transverse and longitudinal [24]. GNRs are significantly small, approximately 85 nm in length and 20 nm in width, and demonstrate good optical tunability by controlling the aspect ratio (AR, length/width) of the NPs [27]. In addition to the seed-mediated growth method as a chemical strategy, physical approaches based on thermal and ultrafast laser-induced heating have also been suggested to modify the morphology of calcinated GNRs [28,29]. Therefore, protecting GNRs from heat-induced shape deformation is essential for effectively using them in particular applications including PTT [29]. Zinc (Zn) is a trace element present in the hard tissues of teeth, muscles, bones, and skin [30]. Zinc oxide (ZnO) is an outstanding material for coating applications owing to its unique properties such as a large surface area, ease of synthesis, easy tunability, safety, controllable size, low cost, and high photocatalytic activity [31]. Zinc oxide (ZnO) has been recently proposed as a nanophotosensitizer for PDT owing to its ability to induce ROS production under light irradiation [32,33]. ZnO was successfully coated on the surface of GNRs via Zn^2+^ precursor hydrolysis in a basic environment [33,34]. Gold nanorod/zinc oxide (GNR@ZnO) core–shell nanocomposites with photothermal and photodynamic properties have been designed for mild PTT-enhanced PDT owing to their excellent biocompatibility, tunable size, large surface area, photothermal stability, photothermal conversion efficiency, and low cost [33]. 3-aminopropyltriethoxysilane (APTES) is an effective salinization coupling agent that was successfully conjugated onto the surface of GNR@ZnO core–shell nanocomposites to form aminated GNR@ZnO-APTES [35]. Mitochondria play an essential role in cells that undergo various changes during apoptosis [36]. Therefore, mitochondrial targeting has emerged as a popular supplementary technique for enhancing the therapeutic efficacy in cancer treatment [37]. Triphenylphosphonium has been extensively employed as a lipophilic cationic agent that can effectively target mitochondria, which have a poor stability [38]. Aminated GNR@ZnO-APTES was effectively functionalized with (4-carboxybutyl) triphenylphosphonium bromide (CTPP) to improve its stability and therapeutic efficacy [37,39]. More importantly, the use of triphenylphosphonium-functionalized gold nanorod/zinc oxide core–shell nanocomposites (CTPP-GNR@ZnO) as all-in-one nanoplatforms has gained considerable research attention for mitochondrial-targeted phototherapy owing to their photothermal conversion efficiency, highest ROS generation, and high mitochondrial-targeting capability.

In this study, GNRs were first synthesized by the seed-mediated growth method, which was then layered with ZnO via the Zn^2+^ precursor hydrolysis in a basic environment, generating highly monodisperse GNR@ZnO core–shell nanocomposites. Moreover, GNR@ZnO core–shell nanocomposites were calcinated in a furnace at various temperatures including 200, 250, 300, and 400 °C for 1 h. APTES was conjugated on the surface of GNR@ZnO (300 °C) core–shell nanocomposites and the aminated GNR@ZnO-APTES was functionalized with a carboxyl group of mitochondrial-targeting CTPP using carbodiimide chemistry to form CTPP-GNR@ZnO core–shell nanocomposites. In addition, the CTPP-GNR@ZnO core–shell nanocomposites exhibited a uniform morphology, photothermal heat conversion efficiency, ROS generation, and mitochondrial-targeting abilities. The results of the CT-26-cell experiment demonstrated that the CTPP-GNR@ZnO core–shell nanocomposites exhibited excellent biocompatibility and outstanding cancer therapy in vitro. Therefore, the CTPP-GNR@ZnO core–shell nanocomposites can be applied as an all-in-one nanoplatform for mitochondrial-targeted phototherapy.

## 2. Materials and Methods

### 2.1. Materials

Benzyldimethylhexadecylammonium chloride (BDAC), gold(III) chloride trihydrate (HAuCl_4_·3H_2_O, 99.9%), cetyltrimethylammonium bromide (CTAB), silver nitrate, _L_-ascorbic acid (AA), sodium borohydride, zinc nitrate (Zn(NO_3_)_2_.6H_2_O, 24 mM), zinc oxide, hexamethylenetetramine (HMT), 1-ethyl-3-(3-dimethylaminopropyl) carbodiimide (EDC), 3-aminopropyltriethoxysilane (APTES), (4-carboxybutyl) triphenylphosphonium bromide (CTPP), N-hydroxysuccinimide (NHS), sodium hydroxide (NaOH), 3-(4,5)-dimethylthiahiazo-2-yl-2,5-diphenyltetrazolium bromide (MTT), calcein-AM, 2′,7′-dichlorodihydrofluorescein diacetate (H_2_DCFDA), 1,3-diphenylisobenzofuran (DPBF), and propidium iodide (PI) were all bought from Sigma-Aldrich (St. Louis, MO, USA) and were used without any purification.

### 2.2. Preparation of CTPP-GNR@ZnO Core–Shell Nanocomposites

The GNRs were synthesized according to previously reported procedures [25,26]. The as-prepared CTAB-capped GNRs were purified by repeated centrifugation and dispersed in 20 mL of deionized (DI) water. For the surface modification of CTAB-capped GNRs, ZnO was layered on the surface of the GNR to form GNR@ZnO core–shell nanocomposites as previously described (with slight modifications), which were synthesized by the Zn^2+^ precursor hydrolysis in a basic environment [33,34]. The growth solution containing (5 mL of each solution) CTAB (24 mM), _L_-ascorbic acid, (12 mM), Zn(NO_3_)_2_.6H_2_O (24 mM), and HMT (24 mM) was gently added in a volume ratio of (1:1:1:1). Purified GNR solution (5 mL) was mixed with 20 mL of growth solution while stirring, and the reaction solution was placed in an oil bath at 80 °C for 6 h. Finally, the GNR@ZnO core–shell nanocomposites were purified by repeated centrifugation and finally dispersed in ethanol for further use. Additionally, GNR@ZnO core–shell nanocomposites were placed in an oven at 60 °C for 12 h, which were then calcinated in a furnace at different temperatures including 200, 250, 300, and 400 °C for 1 h (10 °C/min), obtaining their powdered form for further experiments. For amine conjugation on the surface of the GNR@ZnO core–shell nanocomposites, 400 µL of APTES was added to 10 mL of GNR@ZnO core–shell nanocomposites (300 °C, 1 mg/mL), which was stirred for 4 h at room temperature; the reaction solutions were separated by centrifugation, washed with deionized water, and then aminated GNR@ZnO-APTES was dispersed in deionized water for further uses. For the functionalization of (4-carboxybutyl) triphenylphosphonium bromide (CTPP) on the surface of GNR@ZnO-APTES, the carboxyl group of CTPP was functionalized with the primary amino group of GNR@ZnO-APTES (300 °C) using EDC/NHS chemistry [37,39]. CTPP (10 mg/mL) was dispersed in DI water, and the mixture solution was adjusted to a pH of 5.5 with a 0.1 M NaOH solution. A total of 0.22 g of EDC (46 mM) and 0.011 g of NHS (20 mM) were added to the solution above while stirring for 2 h to activate the carboxyl group of CTPP; the mixture solution was then adjusted to a pH of 8.0. Subsequently, 10 mL of GNR@ZnO-APTES (300 °C) was added to the CTPP solution and the mixture was stirred at room temperature for 1 h. The final product was purified by repeated centrifugation to obtain the CTPP-GNR@ZnO core–shell nanocomposites. 

### 2.3. Characterization of CTPP-GNR@ZnO Core–Shell Nanocomposites

The optical absorption properties of the samples were measured using a UV-2600 spectrophotometer (Shimadzu, Tokyo, Japan). The structure, composition, particle size range, and surface charge of the samples were characterized using high-resolution transmission electron microscopy combined with a selected area electron diffraction pattern (SAED) analysis (HRTEM; JEM-ARM200F, JEOL, Japan), high-angle annular dark-field scanning transmission electron microscopy (HAADF-STEM; JEOL, JEM-2100) with energy-dispersive X-ray (EDX) spectroscopy, and a 90Plus particle size and zeta analyzer (Brookhaven, NY, USA). The samples were also studied using powder X-ray diffraction (XRD; SWXD-D-MAX/2500-PC, Japan) patterns, Fourier-transform infrared spectroscopy (FTIR; Thermo Scientific Nicolet iS5, Waltham, MA, USA), and atomic absorption spectroscopy (AAS; MegaA-700FG, Korea) measurements. Thermogravimetry and a differential thermal analysis (TG/DTA) of the CTPP-GNR@ZnO core–shell nanocomposites were performed using the TGA-500 instrument (Thermo Fisher Scientific, Waltham, MA, USA).

### 2.4. Evaluation of ROS Generation by the DPBF Assay

DPBF was used as a quantitative measurement technique to evaluate the potential of the CTPP-GNR@ZnO (300 °C) core–shell composites to generate ROS under laser irradiation at 780 nm [40]. Briefly, CTPP-GNR@ZnO (300 °C) core–shell nanocomposites and ZnO, at the same concentration of 10 µg Au/mL, were obtained in a 1 cm quartz cuvette along with DPBF (20 µL) mixed in ethanol (10 mmol/L). The initial absorbance was recorded at 410 nm using a Shimadzu UV-2600 spectrophotometer. All the samples were exposed to a 780 nm NIR laser (1.2 W/cm^2^) for 600 s, and the optical density was recorded at 410 nm. 

### 2.5. Photothermal Evaluation

To evaluate the photothermal conversion efficiency, the GNR, GNR@ZnO (300 °C), and CTPP-GNR@ZnO (300 °C) core–shell composites solutions at different concentrations (0, 5, 10, 15, 20, and 30 µg Au/mL) were irradiated with a laser (780 nm, 1.2 W/cm^2^) for 1200 s. The temperature changes were monitored using a CX320 infrared (IR) thermal camera (COX, Korea). Subsequently, the laser was turned off and the solution was gradually returned to room temperature, which was repeated three times to evaluate their photothermal stability. The photothermal conversion efficiency (*η*) of the CTPP-GNR@ZnO core–shell nanocomposites (10 µg Au/mL) can be calculated by the following equation [41,42]:

$$\eta =\frac{hS\left(T_{Max}-T_{Sur}\right)-Q_{dis}}{I(1-10^{-A_{780}})}$$

### 2.6. In Vitro Cytotoxicity

The murine colorectal carcinoma cell line (CT-26) was purchased from the Korean Cell Line Bank (KCLB, Korea). The CT-26 cells were seeded at a density of 6 × 10^4^ cells/well in Dulbecco’s modified Eagle’s medium (DMEM; Welgene, Deagu, Korea) containing 10% of fetal bovine serum (FBS; Welgene) and 1% of penicillin-streptomycin (Welgene) in a humidified atmosphere with 5% CO_2_. The in vitro cytotoxicity was evaluated using the MTT assay. The CT-26 cells were precultured overnight in a 96-well culture plate. Subsequently, the GNR, GNR@ZnO (300 °C), and CTPP-GNR@ZnO (300 °C) core–shell nanocomposites with various concentrations (0–30 µg Au/mL) were added and incubated for 12 h. The percentage of cell viability was finally determined at 570 nm using an ELISA plate reader (BioTek, Winooski, VT, USA). 

### 2.7. In Vitro Cellular Update Evaluation 

To further assess the cellular uptake efficacy of the nanomaterials, the cells were cultured overnight in a cell culture plate at a density of 6 × 10^4^ cells/well. The CT-26 cells were treated with the culture medium containing the GNR, GNR@ZnO (300 °C), and CTPP-GNR@ZnO (300 °C) core–shell nanocomposites at the same concentration (10 µg Au/mL) for 12 h. Subsequently, the cells were collected and fully digested with aqua regia solution (HCl: HNO_3_ = 3:1), and the gold (Au) content was determined using AAS. 

### 2.8. In Vitro Photothermal Therapy

The in vitro photothermal effect was assessed using an MTT assay. The CT-26 cells were cultured overnight in a 48-well plate at a density of 6 × 10^4^ cells/well. The cells were treated with the GNR, GNR@ZnO (300 °C), and CTPP-GNR@ZnO (300 °C) core–shell nanocomposites at the same concentration (10 µg Au/mL) for 6 h, and the plates were irradiated by a 780 nm laser at 1.2 W/cm^2^ for various time periods (0, 600, 1800, and 3000 s). Subsequently, the CT-26 cells were incubated for an additional 4 h, and the optical density was recorded on a microplate reader at 570 nm. The in vitro photothermal effect was further assessed using live/dead cell staining. The CT-26 cells were precultured overnight in a 96-well culture plate at a density of 6 × 10^4^ cells/well, which was followed by the GNR, GNR@ZnO (300 °C), and CTPP-GNR@ZnO (300 °C) core–shell nanocomposites at the same concentration (10 µg Au/mL) being treated and incubated for 6 h. The plates were irradiated with a 780 nm laser at 1.2 W/cm^2^ for various time periods (0, 600, 1800, and 3000 s). After incubation for an additional 4 h, the medium was replaced with PBS containing calcein-AM and PI for 30 min, and images were captured using a confocal laser microscope (A1R, Nikon, Tokyo, Japan) in the tile scan mode of live/dead cells. 

### 2.9. Intracellular ROS Generation

For PDT-induced intracellular ROS generation, the CT-26 cells were incubated with the GNR, GNR@ZnO (300 °C), and CTPP-GNR@ZnO (300 °C) core–shell nanocomposites at the same concentration (10 µg Au/mL) for 6 h and exposed to a 780 nm laser at 1.2 W/cm^2^ for different time periods (0, 600, 1800, and 3000 s). After incubation for an additional 4 h, H_2_DCFDA (10 µM) was added to stain all the cells for 30 min, which was extensively used as a fluorescent probe to identify PDT-stimulated ROS generation. The cells were then observed using a confocal laser microscope in the tile scan mode.

### 2.10. Mitochondrial-Targeting Localization of CTPP-GNR@ZnO Core–Shell Nanocomposites in Cells

The CT-26 cells were cultured overnight at a density of 0.5 × 10^5^ per 35 mm glass-bottom culture plate and were treated with CTPP-GNR@ZnO core–shell nanocomposites (10 µg Au/mL) for 6 h. The plates were irradiated by a 780 nm laser at 1.2 W/cm^2^ for 300 s. After incubation for an additional 4 h, rhodamine B (10 µg/mL) and MitoTracker green (10 µg/mL) were stained for 30 min and images of the cells were obtained by confocal laser scanning microscopy. 

### 2.11. Statistical Analysis

Statistical analyses were performed using the SPSS 23.0 software. Statistical significance was considered at * *p* < 0.05 and ** *p* < 0.01.

## 3. Results and Discussion

### 3.1. Preparation and Characterization of CTPP-GNR@ZnO Core–Shell Nanocomposites

**Scheme 1:**
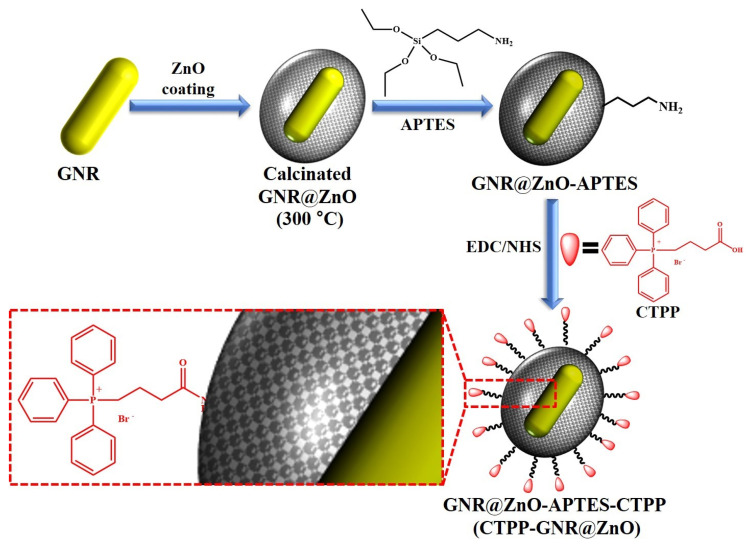
Schematic representation of the preparation procedure of CTPP-GNR@ZnO core–shell nanocomposites.

In this study, GNRs were synthesized using a seed-mediated growth approach [25,26]. The surfaces of the GNRs were layered with ZnO, as previously described, to generate highly monodisperse GNR@ZnO core–shell nanocomposites [33,34]. APTES is an amino-silane linker commonly used in the surface modification of GNR@ZnO (300 °C) core–shell nanocomposites. The aminated GNR@ZnO-APTES (300 °C) was functionalized with a carboxyl group of a mitochondrial-targeting small molecule, such as CTPP, using EDC/NHS chemistry to form CTPP-GNR@ZnO (300 °C) core–shell nanocomposites (Scheme 1) [37,39]. 

**Figure 1:**
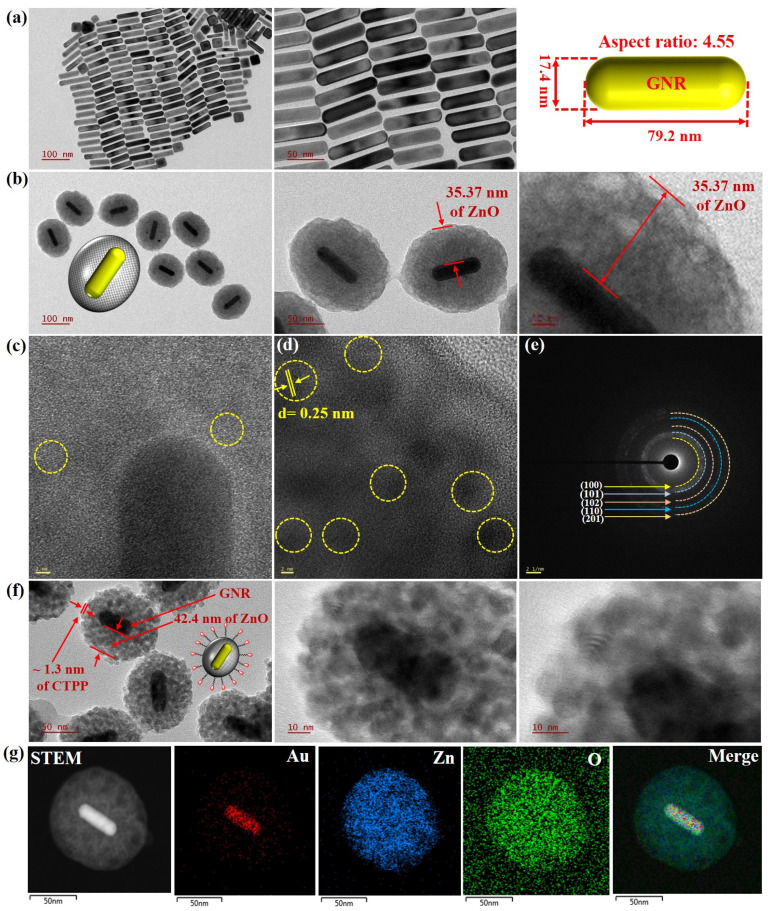
HRTEM images of GNRs (**a**), GNR@ZnO (**b**), with the lattice fringes (yellow circles) of ZnO QDs (**c**,**d**). (**e**) SAED pattern of the ZnO layer. (**f**) HRTEM images of CTPP-GNR@ZnO (300 °C) core–shell nanocomposites. (**g**) STEM-related EDX mapping of CTPP-GNR@ZnO (300 °C) core–shell nanocomposites.

The morphology and size of GNRs, GNR@ZnO, and CTPP-GNR@ZnO core–shell nanocomposites were observed by HRTEM, demonstrating that GNRs exhibit a good uniformity with an average length of 79.2 ± 3.4 nm and an average width of 17.4 ± 1.3 nm, resulting in an aspect ratio of 4.55 (Figure 1a). HRTEM images of GNRs demonstrating the thickness of the ZnO shell are shown in Figure 1b, confirming the formation of a uniform ZnO coating, with averaged shell thicknesses of 35.37 ± 5.3 nm at 200 °C and 42.4 ± 6.7 nm at 300 °C because the crystal size of the ZnO layer was gradually increased at 300 °C. The ZnO layer was composed of numerous ZnO quantum dots (QDs) in the amorphous residue, which were approximately 5 nm in size (Figure 1c). The lattice structures displayed in Figure 1d clearly reveal that the particles were crystalline. The distance between neighboring lattices was determined to be 0.25 nm [43,44]. Figure 1e presents the SAED pattern of ZnO, demonstrating rings corresponding to the (100), (101), (102), (110), and (201) reflections of the face-centered cubic (fcc) structure of ZnO. After CTPP was grafted on the surface of the GNR@ZnO core–shell nanocomposites, they demonstrated a uniform morphology with an average thickness of 1.3 nm, indicating the successful conjugation of CTPP on their surface (Figure 1f). The morphology of the CTPP-GNR@ZnO core–shell nanocomposites was further investigated using HAADF-STEM. The STEM and EDX mapping of the CTPP-GNR@ZnO core–shell nanocomposites (Figure 1g) demonstrates their uniform distribution. 

**Figure 2:**
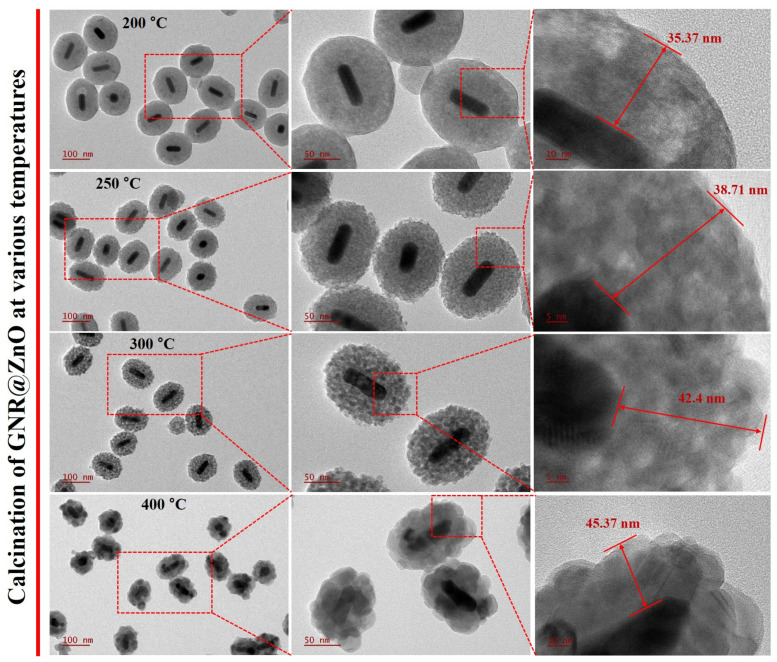
HRTEM images of the calcinated GNR@ZnO at various temperatures (200, 250, 300, and 400 °C).

**Figure 3:**
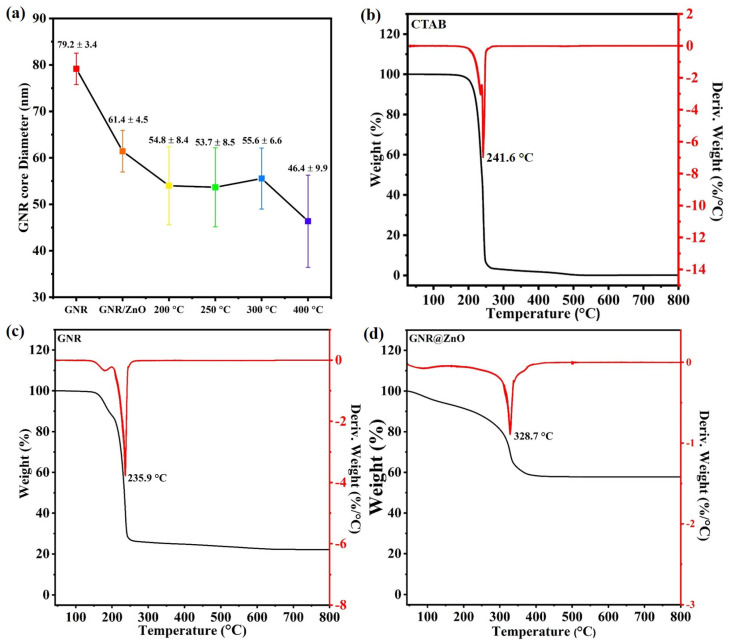
(**a**) The average length of the GNR core varies depending on the calcination temperature of GNR@ZnO. TG/DTA of the CTAB (**b**), GNR (**c**), and GNR@ZnO (**d**). TG corresponds to the black line and DTA corresponds to the blue line.

Calcination is a frequently employed procedure for removing residual surfactants from porous materials templated with surfactants [45]. Calcination temperature is one of the most important factors influencing the morphology and size of nanomaterials [46]. The calcination of GNR@ZnO core–shell nanocomposites was investigated at various temperatures including 200, 250, 300, and 400 °C for 1 h. Figure 2 presents the HRTEM images of the calcinated GNR@ZnO core–shell nanocomposites at 200, 250, 300, and 400 °C. The crystal size of the ZnO layer gradually increased to 35.37 ± 5.3 nm, 38.71 ± 3.8, 42.4 ± 6.7 nm, and 45.37 ± 4.3 nm at 200, 250, 300, and 400 °C, respectively. However, the average length of the GNR core gradually decreased by the calcination procedure and significantly reduced to 46.4 ± 9.9 nm at 400 °C (Figure 3a). Therefore, GNR@ZnO was calcinated for less than 1 h. Previously, we reported that the halide complexes (for example, HX and CH_3_X, X = Br^−^ and Cl^−^) produced from the residual surfactant (CTAB) may dissolve the GNR by the formation of gold–halide complexes during the heat treatment procedure above 300 °C [26]. The thermal stabilities of the CTAB, CTAB-capped GNR, and GNR@ZnO core–shell nanocomposites were further investigated by thermogravimetric and differential thermal analysis (TG/DTA) (Figure 3b–d). The TG/DTA curves of the CTAB and GNR were stable up to 230 °C. The decomposition between 230 °C and 242 °C corresponds to the degradation of the capping agent CTAB, which prevents uncontrolled growth and agglomeration of the GNR [47]. In contrast, the TG/DTA curves of the GNR@ZnO core–shell nanocomposites demonstrated a high thermal stability up to 320 °C. Thermal decomposition appears to occur between 320 and 330 °C, which corresponds to the decomposition of the GNR@ZnO core–shell nanocomposites [48]. 

**Figure 4:**
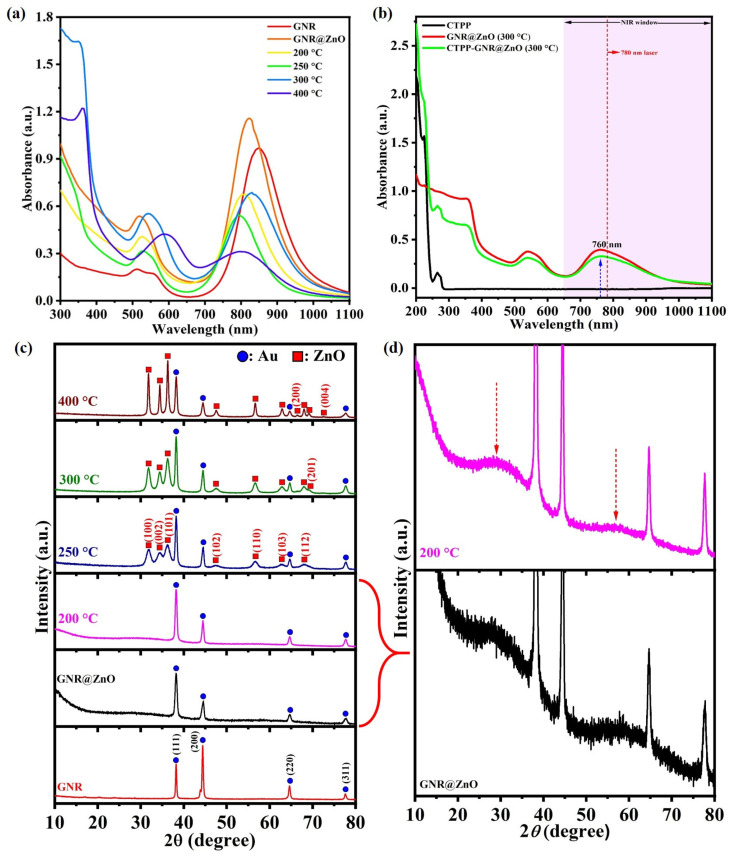
(**a**,**b**) UV–vis spectra of the GNR, GNR@ZnO at various temperatures (200, 250, 300, and 400 °C), CTPP, and CTPP-GNR@ZnO (300 °C) core–shell nanocomposites. (**c**) XRD patterns of the synthesized GNR and GNR@ZnO (200, 250, 300, and 400 °C) core–shell nanocomposites. (**d**) XRD patterns of freshly synthesized GNR@ZnO and GNR@ZnO (200 °C) core–shell nanocomposites (enlarged).

The optical properties of the GNR, GNR@ZnO at various temperatures (200, 250, 300, and 400 °C), CTPP, and CTPP-GNR@ZnO core–shell nanocomposites (300 °C) were further characterized by a Shimadzu UV-2600 spectrophotometer (Figure 4a,b). The UV–vis spectrum of the GNR demonstrated transverse and longitudinal LSPR peaks with maximum absorptions at 510 nm and 850 nm, respectively. For the surface modification of the GNR, the UV–vis absorption spectra of the GNR@ZnO core–shell nanocomposites demonstrated a slightly blue-shifted LSPR peak with respect to that of the GNR. The GNR@ZnO core–shell nanocomposites were heated at various temperatures (200, 250, 300, and 400 °C), demonstrating a significant blue shift of the LSPR peak from 200 to 400 °C. By increasing the calcination temperature, the average length of the GNR core significantly decreased in the LSPR peak, as confirmed by morphological and optical characterization [29]. After conjugation with CTPP, the UV–vis absorption spectra of the CTPP-GNR@ZnO core–shell nanocomposites revealed a distinctive CTPP peak at 265 nm and a significant LSPR peak at 760 nm, suggesting that the mitochondrial-targeting CTPP molecule was efficiently conjugated on the surface of the GNR@ZnO core–shell nanocomposites, which is a highly efficient NIR phototherapy agent owing to its highly efficient capacity to generate photothermal heat and ROS [49]. 

Figure 4c demonstrates the XRD patterns of the synthesized GNR and GNR@ZnO (200, 250, 300, and 400 °C) core–shell nanocomposites. The XRD pattern of the GNR had four distinct features at 2θ = 38.1, 44.4, 64.6, and 77.6, which can be attributed to the strongest line reflections from the (111), (200), (220), and (311) planes of the face-centered cubic (fcc) structure of gold (JCPDS 04-0784) [50]. The GNR@ZnO and calcinated GNR@ZnO (200 °C) demonstrated two distinctive peaks at 29.6 and 56.8 (indicated by the red arrows in the enlarged XRD patterns) owing to the absence of the long-range order in the ZnO crystal; the major peaks of the GNR also appeared at 38.1, 44.4, 64.6, and 77.6, which confirmed the successful formation of the GNR@ZnO core–shell nanocomposites (Figure 4d) [51]. The XRD peaks corresponding to the typical ZnO crystal began to emerge in the calcinated GNR@ZnO above 250 °C. Upon increasing the calcination temperature, the XRD intensity of the ZnO crystals became progressively narrower and stronger, resulting in improved crystallinity. The calcinated GNR@ZnO (250, 300, and 400 °C) demonstrated major characteristic peaks at *2*θ = 31.8, 34.2, 36.3, 47.4, 56.6, 62.7, 68.1, and 69.85, which corresponds to the (100), (002), (101), (102), (110), (103), (112), and (200) crystalline planes of ZnO (JCPDS 36-1451), and also presented four distinct peaks of the GNR, suggesting that ZnO was effectively layered with the GNR [33]. 

**Figure 5:**
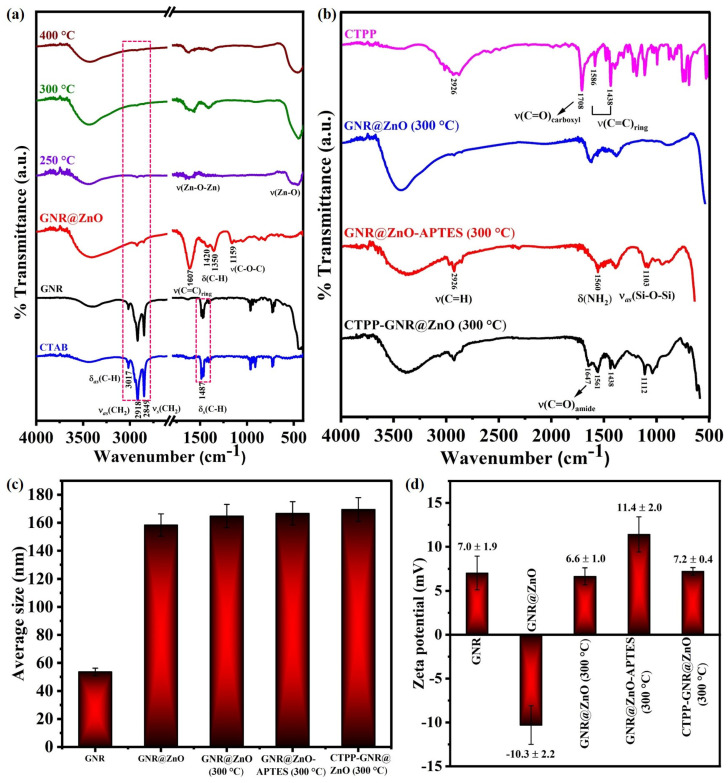
(**a**,**b**) FTIR spectrum of the pure CTAB, CTAB-capped GNR (GNR), GNR@ZnO (RT, 250, 300, and 400 °C), GNR@ZnO-APTES, free CTPP, and CTPP-GNR@ZnO core–shell nanocomposites. (**c**) The average particle size distribution of the GNR, GNR@ZnO, GNR@ZnO (300 °C), GNR@ZnO-APTES (300 °C), and CTPP-GNR@ZnO (300 °C) core–shell nanocomposites. (**d**) Zeta potential of the GNR, GNR@ZnO, GNR@ZnO (300 °C), GNR@ZnO-APTES (300 °C), and CTPP-GNR@ZnO (300 °C) core–shell nanocomposites.

The FTIR spectra of the pure CTAB, CTAB-capped GNR (GNR), GNR@ZnO (250, 300, and 400 °C), GNR@ZnO-APTES, free CTPP, and CTPP-GNR@ZnO core–shell nanocomposites were obtained (Figure 5a,b). The pure CTAB and GNR core demonstrated a strong absorption at 1487, 2849, 2918, and 3017 cm^−1^, which can be assigned to the symmetrical (*δ_s_* (C–H)), (*ν_s_* (CH_2_)), and asymmetrical stretching (*ν_as_* (CH_2_)) of the CH_2_ units, and the asymmetrical bending (*δ_as_* (C–H)) vibrations of the C–H unit in CTAB, because CTAB strongly binds to the surface of the GNR [52,53,54]. After surface modification of the GNR with ZnO, the new vibrational peaks of ZnO were present at 1159 and 1607 cm^−1^, corresponding to the C–O–C stretching (*ν* (C–O–C)) and C=C stretching (*ν* (C=C)_ring_) of the ascorbic acid, respectively. The two strong peaks of CTAB in the GNR weakened, and a major characteristic peak disappeared at 1487 and 3017 cm^−1^, corresponding to the symmetrical (*δ_s_* (C–H)) and asymmetrical bending (*δ_as_* (C–H)) vibrations of the C–H unit for the –(CH_3_)_3_N^+^ head group of CTAB, suggesting that ZnO was efficiently layered on the surface of the GNR core [34,55]. Yang et al. reported that the ascorbic acid (AA) can trap CTAB molecules and subsequently transform them into CTAB-[AA-Zn(OH_4_)]^2−^ complexes during the synthetic procedure for the ZnO layer coating on various metal NPs [34]. The ZnO layer of the GNR@ZnO consisted of the ZnO quantum dots (QDs) and CTAB-[AA-Zn(OH_4_)]^2−^ compounds. Meanwhile, the *ν_s_* (CH_2_) and *ν_as_* (CH_2_) modes for the calcinated GNR@ZnO gradually disappeared as the temperature increased to above 300 °C owing to the elimination of the CTAB-[AA-Zn(OH_4_)]^2−^. Based on this, we confirmed that the optimal calcination temperature for the GNR@ZnO core–shell nanocomposites was approximately 300 °C owing to the excellent crystallinity of the ZnO layer, the fact that the changes in the average length of the GNR were nearly identical, and the fact that the cytotoxic CTAB surfactant was successfully removed. For the conjugation of amine (APTES) on the surface of the GNR@ZnO (300 °C) core–shell nanocomposites, APTES in the GNR@ZnO core–shell nanocomposites clearly exhibited typical vibration peaks at 1560 and 1103 cm^−1^, corresponding to the bending vibration of the –NH_2_ group (δ (NH_2_)) and the asymmetrical stretching of Si–O–Si (*ν_as_* (Si-O-Si)), respectively. Furthermore, the strong characteristic peaks of GNR@ZnO appeared, suggesting that APTES was successfully crosslinked on the surface of the GNR@ZnO core–shell nanocomposites [56,57,58]. For CTPP functionalization on the surface of GNR@ZnO-APTES, CTPP revealed strong peaks at 1438, 1586, and 1708 cm^−1^, indicating the C=C stretching peak (ν (C=C)_ring_) of the aromatic ring and C=O stretching vibration (ν (C=O)_carboxyl_) of the carboxyl group, respectively [59]. The strong absorption bands at 1438 cm^−1^ in the CTPP-GNR@ZnO (300 °C) core–shell nanocomposites were attributed to the C=C stretching peak (ν (C=C)_ring_) of the aromatic ring. Other absorption bands of CTPP were present in the FTIR spectra of CTPP-GNR@ZnO, demonstrating slight shifts from 1561 to 1647 cm^−1^, corresponding to the ν (C=O)_carboxyl_ peak owing to the formation of the amide bond (ν (C=O)_amide_) and C=C stretching peak (ν (C=C)_ring_) of the aromatic ring, respectively. Most absorption peaks of GNR@ZnO were also present, suggesting that CTPP was successfully conjugated on the surface of the GNR@ZnO core–shell nanocomposites through EDC/NHS coupling to form an amide bond [60,61]. 

To further investigate the size and distribution of the GNR, GNR@ZnO, GNR@ZnO (300 °C), GNR@ZnO-APTES (300 °C), and CTPP-GNR@ZnO (300 °C) core–shell nanocomposites, dynamic light scattering (DLS) was performed, which demonstrated a more uniform particle size distribution with an average diameter of 53.57 ± 2.67 nm, 158.36 ± 5.9 nm, 164.81 ± 6.24 nm, 166.74 ± 5.33 nm, and 169.47 ± 4.47 nm, respectively (Figure 5c). Measurement of the zeta potential revealed that the GNR had a positive charge (7.0 ± 1.9 mV) owing to a cationic surfactant (CTAB) on the surface of the GNR and GNR@ZnO had a negative charge (−10.3 ± 2.2 mV) owing to the CTAB-[AA-Zn(OH_4_)]^2−^ complex in the ZnO layer. The zeta potential of the GNR@ZnO (300 °C) core–shell nanocomposites was positively shifted (6.6 ± 1.0 mV) owing to the formation of hydroxylated [Zn(OH)]^+^ species on the surface of the ZnO crystal at a neutral pH [62]. For the APTES and CTPP conjugation on the surface of GNR@ZnO (300 °C) core–shell nanocomposites, the zeta potentials of GNR@ZnO-APTES (300 °C), and CTPP-GNR@ZnO (300 °C) core–shell nanocomposites increased to 11.4 ± 2.0 mV and 7.2 ± 0.4 mV, respectively, verifying the successful modification of the GNR@ZnO core–shell nanocomposites (Figure 5d). 

### 3.2. Evaluation of ROS Generation by DPBF Assay

To estimate the generation of ROS by the ZnO and CTPP-GNR@ZnO (300 °C) core–shell nanocomposites, a DPBF assay was performed by monitoring the absorbance of DPBF at 410 nm. The generation of ROS can effectively quench the DPBF, resulting in ROS generation. As shown in Figure S1, ROS production by the CTPP-GNR@ZnO (300 °C) core–shell nanocomposites under a laser irradiation at 780 nm drastically decreased in a time-dependent manner and was slightly lower than that of ZnO, indicating that the 780 nm laser irradiation can effectively enhance the ROS production of CTPP-GNR@ZnO (300 °C) core–shell nanocomposites. 

### 3.3. Photothermal Evaluation 

**Figure 6:**
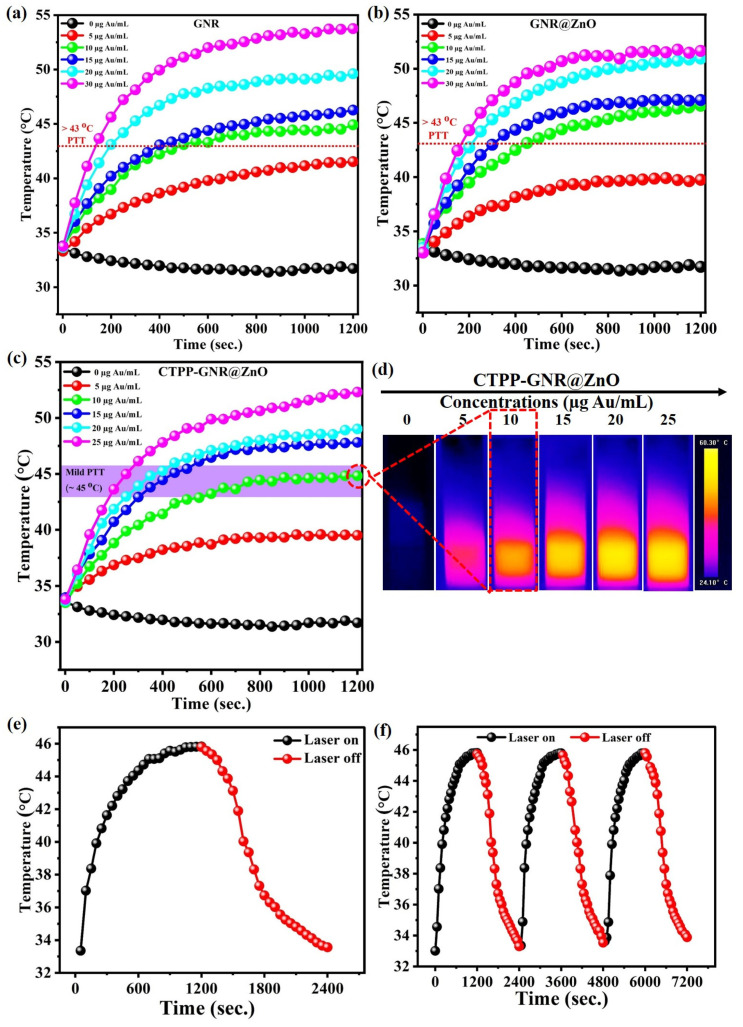
Temperature changes in the GNR (**a**), GNR@ZnO (300 °C) (**b**), and CTPP-GNR@ZnO (300 °C) core–shell composites (**c**) solutions at various concentrations (0, 5, 10, 15, 20, and 30 µg Au/mL) under 780 nm laser irradiation (1.2 W/cm^2^) for 1200 s. (**d**) IR thermal images of the CTPP-GNR@ZnO (300 °C) core–shell composites solutions at various concentrations (0, 5, 10, 15, 20, and 30 µg Au/mL) under 780 nm laser irradiation (1.2 W/cm^2^) for 1200 s. (**e**) Temperature changes in the CTPP-GNR@ZnO (300 °C) core–shell composites (10 µg Au/mL) under 780 nm laser irradiation at 1.2 W/cm^2^ for 1200 s, where the solution gradually returned to room temperature after 1200 s. (**f**) Repeated temperature heating–cooling profiles for three cycles.

The GNRs demonstrated a significant photothermal conversion efficiency, which can produce high temperatures within a short laser irradiation time [63]. The photothermal effect of the GNR, GNR@ZnO (300 °C), and CTPP-GNR@ZnO (300 °C) core–shell composites solutions at various concentrations (0, 5, 10, 15, 20, and 30 µg Au/mL) was evaluated upon 780 nm laser irradiation (1.2 W/cm^2^) for 1200 s, which showed a concentration-dependent temperature change (1.2 W/cm^2^) (Figure 6a–c). Notably, the temperature of the CTPP-GNR@ZnO (300 °C) core–shell composites (10 µg Au/mL) rapidly increased from 33.49 °C to 45.37 °C, where a mild temperature was more attractive and feasible for PTT, whereas it only slightly increased to 31.71 °C for deionized water. The temperature changes in the CTPP-GNR@ZnO (300 °C) core–shell composites (0, 5, 10, 15, 20, and 30 µg Au/mL) were further investigated using a CX320 infrared (IR) thermal camera (Figure 6d). To further assess the photothermal heating conversion efficiency, the temperature changes in the CTPP-GNR@ZnO (300 °C) core–shell composites (10 µg Au/mL) upon irradiation by a 780 nm laser (1.2 W/cm^2^) for 1200 s were evaluated, where the solution gradually returned to room temperature in 1200 s (Figure 6e). A temperature heating–cooling curve was obtained, and the photothermal heating conversion efficiency (*η*) of the CTPP-GNR@ZnO (300 °C) core–shell composites (10 µg Au/mL) was ascertained to be 20.36%. Additionally, the photothermal stability of the CTPP-GNR@ZnO (300 °C) core–shell composites (10 µg Au/mL) was tested and exhibited (Figure 6f), demonstrating no noticeable changes after three on/off cycles of the laser. 

### 3.4. In Vitro Cytotoxicity and Evaluation of Cellular Uptake 

**Figure 7:**
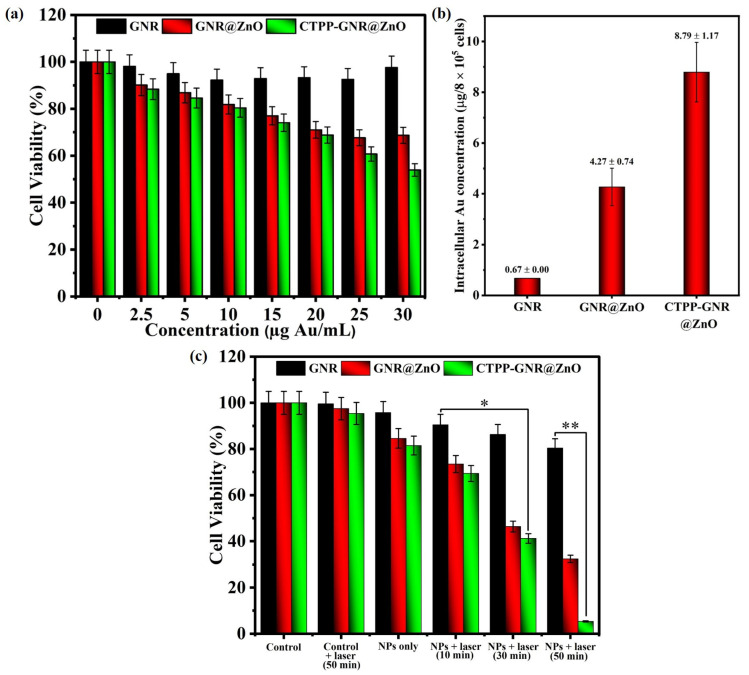
(**a**) Cell viability of CT-26 cells after incubation with different concentrations of the GNR, GNR@ZnO (300 °C), and CTPP-GNR@ZnO (300 °C) core–shell nanocomposites. (**b**) Cellular uptake behavior of the GNR, GNR@ZnO (300 °C), and CTPP-GNR@ZnO (300 °C) core–shell nanocomposites. (**c**) Cell viability of CT-26 cells after the GNR, GNR@ZnO, and CTPP-GNR@ZnO core–shell nanocomposites at the same concentration (10 µg Au/mL) underwent 780 nm laser irradiation at 1.2 W/cm^2^ for various time periods (0, 600, 1800, and 3000 s). The data are expressed as the mean and standard deviations (* significant *p* < 0.05, ** significant *p* < 0.01).

The cytotoxicity of the GNR, GNR@ZnO (300 °C), and CTPP-GNR@ZnO (300 °C) core–shell nanocomposites with various concentrations (0–30 µg Au/mL) were initially tested using the MTT assay. The cell viabilities of the CTPP-GNR@ZnO (300 °C) core–shell nanocomposites (10 µg Au/mL) were greater than 80%, indicating a negligible cytotoxicity and usability as an excellent biocompatible phototherapy agent for cancer therapy (Figure 7a). Additionally, evaluation of the cellular uptake of the GNR (10 µg Au/mL), GNR@ZnO (300 °C, 10 µg Au/mL), and CTPP-GNR@ZnO (300 °C, 10 µg Au/mL) core–shell nanocomposites (10 µg Au/mL) in CT-26 cells was performed by an AAS (Figure 7b). The intercellular uptake of the CTPP-GNR@ZnO core–shell nanocomposites increased, and the amount of Au was determined to be 8.79 ± 1.17 μg Au /mL per 8 × 10^5^ cells at 12 h compared to 0.67 and 4.27 ± 0.74 μg Au/mL per 8 × 10^5^ cells for a nonspecific target GNR and GNR@ZnO, indicating that the CTPP-GNR@ZnO core–shell nanocomposites improved the cellular uptake owing to their higher efficiency of mitochondrial-targeting CTPP, inducing the death of cancer cells [37]. 

### 3.5. In Vitro Photothermal Therapy

Based on their in vitro biocompatibility, the cytotoxic effects of the GNR, GNR@ZnO (300 °C), and CTPP-GNR@ZnO (300 °C) core–shell nanocomposites were studied in vitro. To assess the influence of the photothermal behavior, CT-26 cells were treated with the GNR, GNR@ZnO, and CTPP-GNR@ZnO core–shell nanocomposites at the same concentration (10 µg Au/mL) for 6 h and the plates were irradiated by a 780 nm laser (1.2 W/cm^2^) for various time periods (0, 600, 1800, and 3000 s) (Figure 7c). Notably, the CTPP-GNR@ZnO core–shell nanocomposites (10 µg Au/mL) demonstrated effective cell ablation (95%) after 50 min of laser irradiation at 780 nm, whereas the cell ablation utilizing the GNR and GNR@ZnO was 80% and 32%, respectively. This demonstrated that the CTPP-GNR@ZnO core–shell nanocomposites have the potential to be used as a promising photothermal material for the treatment of cancer, suggesting that the raised temperature can completely ablate cancer cells. 

**Figure 8:**
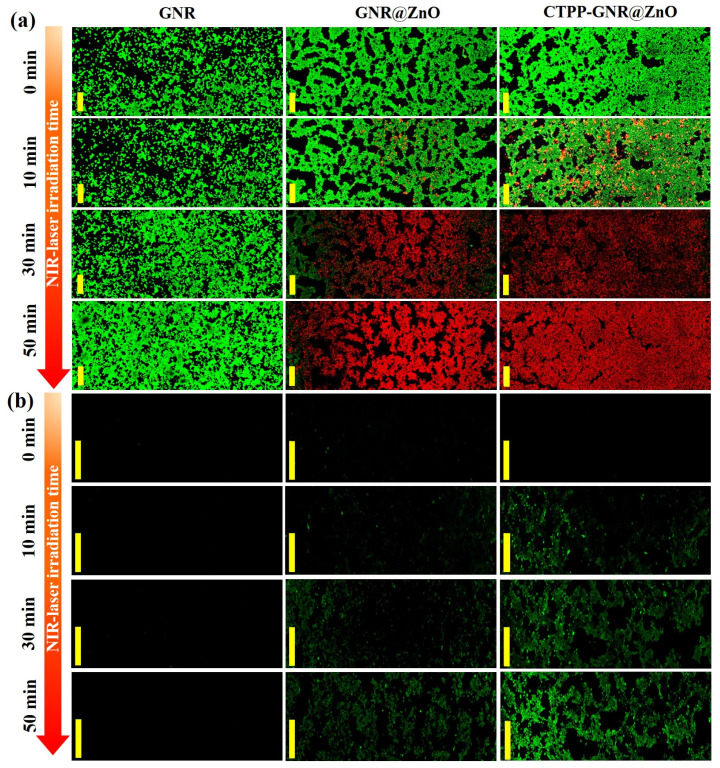
(**a**) The merged confocal fluorescence images of the CT-26 cells after phototherapy treatment of the GNR, GNR@ZnO, and CTPP-GNR@ZnO core–shell nanocomposites at the same concentration (10 µg Au/mL) under 780 nm NIR laser irradiation at 1.2 W/cm^2^ for various time periods (0, 600, 1800, and 3000 s) by conducting a tile scan mode. Calcein-AM (green) and PI (red) were co-stained in these experiments (10× magnification; scale bar: 250 µm). (**b**) H_2_DCFDA staining confocal fluorescence image of the CT-26 cells after being incubated with the GNR, GNR@ZnO, and CTPP-GNR@ZnO core–shell nanocomposites at the same concentration (10 µg Au/mL) under 780 nm NIR laser irradiation at 1.2 W/cm^2^ for various time periods (0, 600, 1800, and 3000 s) by conducting a tile scan mode (10× magnification; scale bar: 250 µm).

The photothermal effect of the GNR, GNR@ZnO, and CTPP-GNR@ZnO core–shell nanocomposites at the same concentration (10 µg Au/mL) was further studied and visualized by live/dead cell fluorescent staining. A large area (1.5 × 2.8 mm^2^) of a fluorescence image is displayed in (Figure 8a), in which several fluorescence image frames were automatically collected. These broad-range tiling images were helpful in tracking the affected region of the entire cell culture area when exposed to NIR laser irradiation. Calcein-AM (green) and PI (red) were co-stained. The CT-26 cells were incubated with the GNR, GNR@ZnO, and CTPP-GNR@ZnO, and the plates were exposed to a 780 nm laser (1.2 W/cm^2^) for different time periods (0, 600, 1800, and 3000 s). A stronger green fluorescence was observed in the CT-26 cells incubated with the control, control + 780 nm laser, GNR + 780 nm laser, GNR@ZnO only, and CTPP-GNR@ZnO only; the amount of red fluorescence observed was negligible. In contrast, the CT-26 cells incubated with CTPP-GNR@ZnO demonstrated a strong red fluorescence after 30 and 50 min of 780 nm laser irradiation, suggesting that CTPP-GNR@ZnO was completely killed after 50 min of laser irradiation. Similar experiments were reported by Seo et al. [49], indicating that the photothermal effect of methylene blue (MB)-loaded mesoporous silica-coated gold nanorods (GNR@mSiO_2_) on graphene oxide (MB-GNR@mSiO_2_-GO) exhibited a stronger red fluorescence after NIR laser irradiation (780 nm) for 3000 s. As another example, Han et al. reported the use of silica-coated GNR (GNR@SiO_2_) for photothermal therapy, which demonstrated that the highest cell death was after laser irradiation for 50 min, indicating that NPs with laser irradiation can effectively induce cell death [26]. 

### 3.6. Intracellular ROS Generation

The PDT-induced generation of intracellular ROS was investigated using H_2_DCFDA, which detected ROS production in live cells [64]. Figure 8b demonstrates that cells treated with GNR did not present a green fluorescence under the 780 nm laser irradiation. Cells treated with the GNR@ZnO core–shell nanocomposites demonstrated a weak green fluorescence after the 780 nm laser irradiation. In contrast, the CT-26 cells incubated with CTPP-GNR@ZnO (300 °C) core–shell nanocomposites exhibited a significant increase in the ROS generation after the 780 nm laser irradiation for 10 min compared to those incubated with the GNR and GNR@ZnO (300 °C). After the 780 nm NIR laser irradiation for 30 min, the resulting ROS level progressively increased to the largest cell culture area for the CTPP-GNR@ZnO (300 °C)-treated CT-26 cells, indicating that CTPP-GNR@ZnO core–shell nanocomposites can induce ROS production in CT-26 cells using the 780 nm NIR laser irradiation, and the CTPP-mediated mitochondrial targeting promoted the generation of more ROS within cancer cells [65]. 

**Figure 9:**
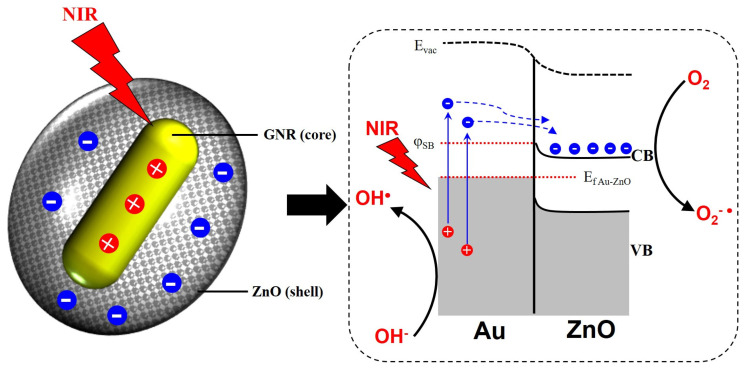
Schematic energy diagrams of GNR@ZnO. A Schottky junction is formed at the interface area between the GNR and ZnO by bending the energy level of ZnO. φ_SB_, E_vac_, E_f Au-ZnO_, CB, and VB represent the Schottky barrier potential, vacuum level energy, fermi level energy, conduction band, and valence band, respectively.

The proposed mechanism of ROS generation by the GNR@ZnO is illustrated in Figure 9. For the GNR@ZnO nanostructure, the work function of Au (φ_Au_ = 5.1 eV) was higher than that of ZnO (φ_ZnO_ = 4.2 eV) as the *n*-type semiconductor, leading to the creation of a Schottky barrier (φ_SB_) at their interface by the downward bending of the energy band of ZnO [66]. Consequently, the charge carriers were easily separated from one another, leading to enhanced ROS generation via the increased photocatalytic activity. Zhou et al. reported that the reduced superoxide (O_2_^−●^) radical species in the ZnO layer can be converted to a singlet oxygen (^1^O_2_) at the GNR core via oxidation [33].

### 3.7. Mitochondrial-Targeting Localization of the CTPP-GNR@ZnO Core–Shell Nanocomposites in Cells

**Figure 10:**
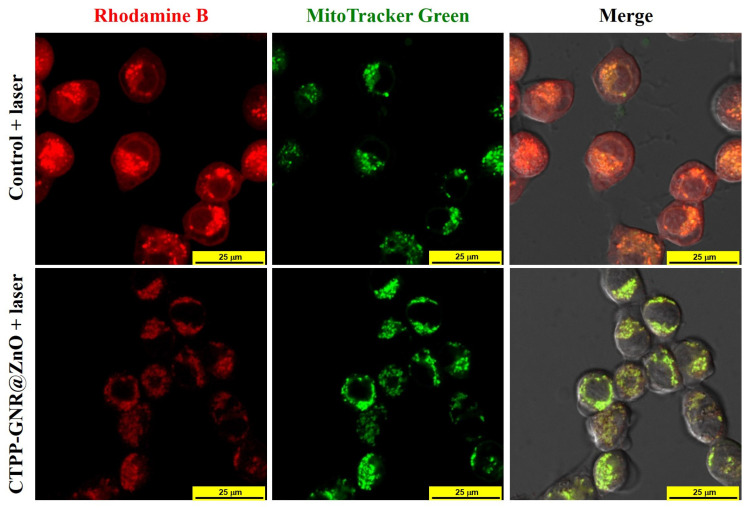
Confocal scanning fluorescence images of CT-26 cells treated with CTPP-GNR@ZnO core–shell nanocomposites with laser irradiation (1.2 W/cm^2^) for 300 s. Rhodamine B and MitoTracker green were co-stained in these experiments (40× magnification; scale bar: 25 µm).

To prove that small molecules such as CTPP have excellent mitochondrial-targeting capabilities for specific positions, confocal fluorescence imaging was employed. The localization and targeting of the CTPP-GNR@ZnO core–shell nanocomposites were further investigated using established mitochondrial-targeting fluorescent probes, such as rhodamine B and MitoTracker green (Figure 10). Rhodamine B is a lipophilic cationic dye that accurately stains the mitochondria, and MitoTracker green is a green fluorescent dye that accurately accumulates in the mitochondria. The CT-26 cells treated with CTPP-GNR@ZnO core–shell nanocomposites demonstrated red fluorescence in the cytoplasm after laser irradiation, which clearly indicated a significant reduction in the mitochondrial membrane potential compared to the control cells. CTPP-GNR@ZnO core–shell nanocomposites located in the cytoplasm co-localized with the mitochondria, confirming that CTPP-GNR@ZnO core–shell nanocomposites can specifically target the mitochondria in CT-26 cells. The red fluorescence of the CTPP-GNR@ZnO core–shell nanocomposites also coincided with the green fluorescence of MitoTracker green, once again confirming that the CTPP-GNR@ZnO core–shell nanocomposites can effectively target the mitochondria in CT-26 cells [36]. 

## 4. Conclusions

In summary, a CTPP-GNR@ZnO core–shell nanocomposite based on NIR-induced ROS production was developed to achieve highly effective mitochondrial-targeted cancer therapy. The CTPP-GNR@ZnO core–shell nanocomposites exhibited outstanding photothermal properties, ROS generation efficiency, broad NIR absorbance, stability, and mitochondrial-targeting efficiency. The CTPP-GNR@ZnO core–shell nanocomposites exhibited significantly low cytotoxic effects against the CT-26 cells without the 780 nm laser irradiation and demonstrated an excellent biocompatibility. Notably, the CTPP-GNR@ZnO core–shell nanocomposites showed effective cell ablation (95%) after the 780 nm NIR laser irradiation for 50 min, indicating that the increased temperature can completely ablate cancer cells. CTPP-GNR@ZnO core–shell nanocomposites also induced a significant amount of intracellular ROS after the 780 nm laser irradiation, suggesting that CTPP-GNR@ZnO core–shell nanocomposites can specifically target the mitochondria of CT-26 cells. Overall, this study provides a new approach for achieving efficient cancer phototherapy, which can become highly valuable in the future.

## Data Availability

Data are contained within the article and supplementary materials.

## Supplementary Materials

The following supporting information can be downloaded at: https://www.mdpi.com/article/10.3390/pharmaceutics16020284/s1.
