# Supplementary material for: Triphenylphosphonium-Functionalized Gold Nanorod/Zinc Oxide Core–Shell Nanocomposites for Mitochondrial-Targeted Phototherapy
Source: Pharmaceutics. 2024 Feb 16;16(2):284. doi: 10.3390/pharmaceutics16020284 (PMC10893051; doi:10.3390/pharmaceutics16020284)
Supplement: Supplementary file 1 [file pharmaceutics-16-00284-s001.zip › pharmaceutics-2832353-supplementary.pdf]

Triphenylphosphonium-Functionalized Gold Nanorod/Zinc Oxide Core–Shell Nanocomposites for Mitochondrial-Targeted Phototherapy

Ara Joe †, Hyo-Won Han †, Yu-Ra Lim, Panchanathan Manivasagan and Eue-Soon Jang *

Department of Applied Chemistry, Kumoh National Institute of Technology, Gumi 730-701, Gyeongbuk, Republic of Korea; jar@kumoh.ac.kr (A.J.); 20101414hyowon@kumoh.ac.kr (H.-W.H.); tig02268@naver.com (Y.-R.L.) manimaribtech@kumoh.ac.kr (P.M.)

* Correspondence: euesoon@kumoh.ac.kr

† These authors contributed equally to this work.

S1. Materials and methods

S1.1. Synthesis of Gold Nanorod (GNR)

Gold nanorods (GNRs) were prepared using the seed-mediated and Ag-catalyzed growth procedure. In brief, a seed solution was prepared by adding cooled $NaBH_4$ (10 mM, 0.6 mL) to a solution of CTAB (20 mM, 5 mL) and $HAuCl_4{\cdot}3H_2O$ (0.5 mM, 5 mL) and vigorously stirring for 3 minutes. The solution was stored separately for over 2 hours at 25 °C. In a mixture of 0.15 M CTAB and 0.02 M BDAC (100 mL), solutions of $HAuCl_4{\cdot}3H_2O$ (1 mM, 100 mL), $AgNO_3$ (4 mM, 5 mL), and AA (79 mM, 1.4 mL) were sequentially added with stirring. The seed solution (0.24 mL) was immediately added to the colorless solution, and the mixture was gently stirred overnight at 30 °C. The produced GNRs were centrifuged at 15,000 rpm for 30 minutes and washed twice with deionized (DI) water to remove excess CTAB. Subsequently, they were dispersed in 20 mL of DI water.

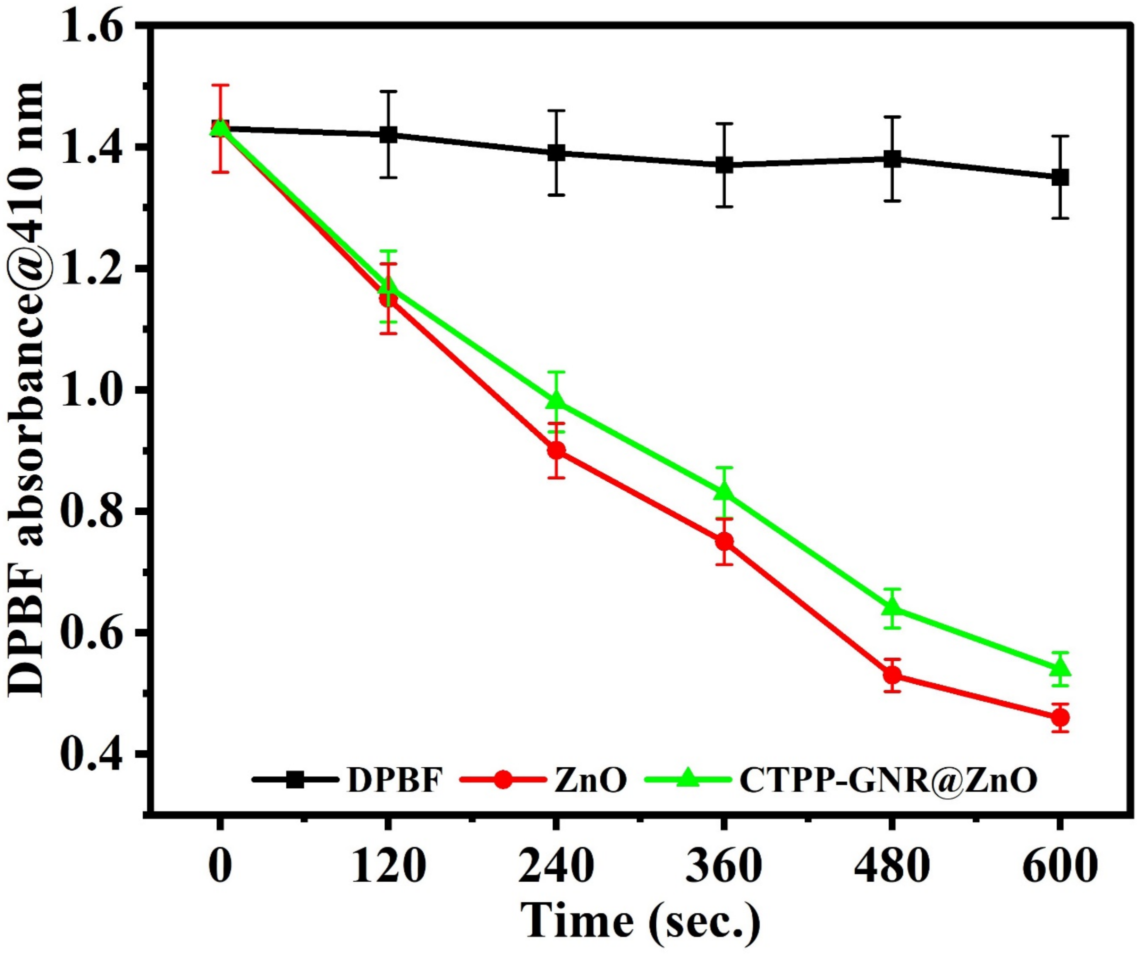

Figure S1. ROS generation ability of ZnO and CTPP-GNR@ZnO core-shell nanocomposites under 780 nm laser (1.2 W/cm^2) for 600 s.
